# Survey on Recommended Health Care for Adult Patients with Myelodysplastic Syndromes Identifies Areas for Improvement

**DOI:** 10.3390/ijerph17249562

**Published:** 2020-12-21

**Authors:** Ioannis Chanias, C. Matthias Wilk, Rudolf Benz, Michael Daskalakis, Georg Stüssi, Adrian Schmidt, Ulrike Bacher, Nicolas Bonadies, on behalf of the Swiss MDS Study Group

**Affiliations:** 1 Department of Hematology and Central Hematology Laboratory, Inselspital, Bern University Hospital, University of Bern, 3010 Bern, Switzerland; ioannis.chanias@insel.ch (I.C.); michael.daskalakis@insel.ch (M.D.); veraulrike.bacher@insel.ch (U.B.); 2 Department of Medical Oncology and Hematology, University Hospital Zurich and University of Zurich, 3010 Bern, Switzerland; Matthias.wilk@mssm.edu; 3 Department of Hematology and Oncology, Cantonal Hospital Muensterlingen, 8596 Muensterlingen, Switzerland; rudolf.benz@stgag.ch; 4 Clinic of Hematology, Oncology Institute of Southern Switzerland, 6500 Bellinzona, Switzerland; Georg.Stuessi@eoc.ch; 5 Clinic for Medical Oncology and Hematology, City Hospital Waid and Triemli, 8063 Zurich, Switzerland; Adrian.Schmidt@triemli.zuerich.ch; 6 Department for BioMedical Research (DBMR), University of Bern, 3010 Bern, Switzerland

**Keywords:** myelodysplastic syndromes, guideline-adherence, survey, areas for improvement

## Abstract

The impact on health care of patients with myelodysplastic syndromes (MDS) is continuously rising. To investigate the perception of hemato-oncologists concerning the recommended MDS patient care in Switzerland, we conducted a web-based survey on diagnosis, risk-stratification and treatment. 43/309 physicians (13.9%) replied to 135 questions that were based on current guidelines between 3/2017 and 2/2018. Only questions with feedback-rates >50% were further analysed and ratios >90% defined “high agreement”, 70–90% “agreement”, 30–70% “insufficient agreement” and <30% “disagreement”. For diagnosis, we found insufficient agreement on using flow-cytometry, classifying MDS precursor conditions, performing treatment response assessment after hypomethylating agents (HMA) and evaluating patients with suspected germ-line predisposition. For risk-stratification, we identified agreement on using IPSS-R but insufficient agreement for IPSS and patient-based assessments. For treatment, we observed disagreement on performing primary infectious prophylaxis in neutropenia but agreement on using only darbepoetin alfa in anaemic, lower-risk MDS patients. For thrombopoietin receptor agonists, insufficient agreement was found for the indication, preferred agent and triggering platelet count. Insufficient agreement was also found for immunosuppressive treatment in hypoplastic MDS and HMA dose adjustments. In conclusion, we identified areas for improvement in MDS patient care, in need of further clinical trials, information, and guiding documents.

## 1. Introduction

Guidelines and recommendations (G/Rs) for the diagnostic work-up and treatment of adult patients with myelodysplastic syndromes (MDS) are published [1,2,3]. However, the level of adherence in the daily clinical practice remains unclear, both in Switzerland and in many other countries. 

MDS represent a heterogeneous group of hematopoietic stem cell disorders with a variable risk of transformation into secondary acute myeloid leukemia (AML). The syndromes are diagnosed at a median age above 70 years and characterized by cytopenias, dysplasia and inflammation [4,5]. Due to aging of the population and the integration of next generation sequencing (NGS) in the diagnostic evaluation of unclear cytopenias, we are observing an increase of MDS cases in recent years. The heterogeneity of the disease, complexity of management and multi-morbidity of the mainly elderly MDS patients leads to an increasing burden on health care systems with the risk for inappropriate use of resources [4,5].

Adherence to G/Rs is generally considered as good quality of care and a cornerstone for appropriate health service [6,7]. Hence, national and international, evidence-based guidelines for diagnosis and therapy of adult MDS have been published, in order to assist clinicians in their daily decision-making [1,2,3]. However, publishing G/Rs does not necessarily translate into better quality of care, if adherence to G/Rs is not implemented and maintained into daily routine [7,8]. Numerous studies have highlighted the difficulties to follow clinical practice guidelines, with non-adherence reaching up to 70% across most disciplines and countries [9,10,11]. Population-based registries and cohorts revealed that around one quarter of MDS patients did not receive the required bone marrow cytogenetics analysis [12,13,14,15,16,17], which impedes appropriate risk calculation by IPSS or IPSS-R. Similarly, the iron status and the endogenous EPO-level were determined in only half of patients receiving erythropoiesis-stimulating agents (ESA) [12,15,18]. This implies that many lower-risk MDS patients receive ESA without complete assessment and estimation of their chance for response. Retrospective studies have shown general shortcomings in adherence to recommended health care procedures [19,20]. Recently, a pro and retrospective analysis of MDS patients treated in a tertiary care center in Germany showed that adherence to G/Rs for allogeneic hematopoietic stem cell transplantation (allo HSCT) resulted in improved survival [21]. However, a systematic investigation of the level of adherence, potential reasons for non-adherence and the impact on other outcomes than survival has not been performed so far.

Here, we surveyed the perception of hemato-oncologists on how appropriate diagnostics, risk-stratification and treatment should be provided to adult MDS patients in Switzerland and compared their feedbacks to currently published G/Rs.

## 2. Methods

### 2.1. Study Design

We performed a survey among physicians between 3/2017 and 2/2018. The survey was initiated by the Swiss MDS Study Group (SMSG), an independent, research consortium of hemato-oncologists practicing in Switzerland. It received funding by grants from public institutions, which did not have any influence on the design and interpretation of the results. Approval from the regional ethics committee was not required.

### 2.2. Participants

Participants were recruited either by e-mail through the SMSG and the Swiss Society of Hematology (SSH) or personally during educational conferences. From 309 hematologists and oncologists registered at SSH, 42 practicing in Switzerland and one in Germany replied to our survey. The characteristics of the participants are summarized in Table 1. Most participants were younger than 50 years (30/43, 70%) and received their board certification after 2000 (29/43, 67%). Three quarters worked in secondary or tertiary centers (32/43, 74%) with 84% (36/43) being members of the SSH and 58% (25/43) of the European Hematology Association (EHA). Most were familiar with ELN 2013 MDS recommendations for diagnosis (38/43, 88.4%) and treatment (37/43, 86%) [1].

### 2.3. Survey Design

The survey was designed on the REDCap system [22]. It was composed of 135 multiple-choice questions covering the whole spectrum of MDS patient care and based on the recommendations from the European Leukemia Net (ELN) 2013 MDS guideline, from 3/2017 until 2/2018 [1]. The questions comprised the three domains of diagnostic evaluation (*n* = 18), risk-stratification (*n* = 12) and treatment, including supportive care (*n* = 21), growth factors (*n* = 22), disease modifying treatment (imids, immune modulating agents, hypomethylating agents) (*n* = 40) and allo HSCT (*n* = 22).

### 2.4. Data Collection

The survey was submitted by e-mail and contained general information on the background, aims and performance of the survey including a link to the web-based survey. From all contacted physicians (*n* = 309), we received a feedback from 43 participants (overall feedback-rate 13.9%).

### 2.5. Data Processing and Evaluation

After closure of the survey, data was directly exported from REDCap. In a first step, we evaluated in how far the feedbacks were representative. From the 43 participants, we defined an optimal feedback-rate >75%, whereas 50–75% was defined as sufficient and <50% insufficient for further analysis. Questions regarding allo HSCT were considered separately, as only six participants were involved in this type of treatment. 41 out of 135 questions (30.4%) had an insufficient feedback-rate and were excluded from further evaluation (Supplementary Materials Table S1). The agreement score for each representative question was determined by the ratio of all participants who selected the corresponding item. We defined >90% as “high agreement”, 70–90% as “agreement”, 30–70% as “insufficient agreement”, and <30% as “disagreement”.

### 2.6. Data Representation and Statistics

We used descriptive statistics for the representation and analysis of results. Discrete variables are presented as absolute and relative numbers, continuous variables are shown as intervals and median values for the comparison between different patient groups.

## 3. Results

### 3.1. Diagnostic Evaluation

We received sufficient feedbacks (>50% of all participants) for 17 of 18 questions (94%) in this domain, while only the question on single gene analysis had to be dropped (Table 2 and Supplementary Materials Table S1). We observed high agreement in using the most recent revision of the World Health Organization 2016 classification system for diagnosis of MDS and its subtypes [23]. In contrast, agreement was insufficient regarding the use of the diagnostic terms of “clonal hematopoiesis of indeterminate potential” (CHIP), “clonal cytopenia of unknown significance” (CCUS), and “idiopathic cytopenia/dysplasia of unknown significance” (ICUS/IDUS (29/43, 67.4%) [24]. There was high agreement on the need of bone marrow (BM) examination at suspected diagnosis (43/43, 100%) and progression (and 40/43, 93%), while insufficient agreement was found on the assessment of response to hypomethylating agents (HMA) (20/43, 46.5%). Participants agreed that the inter-observer variability for the morphological quantification of blasts is acceptable (31/43, 72.1%) but their agreement was insufficient concerning an acceptable inter-observer variability for the morphological assessment of dysplasia (24/43, 55.8%). We found full agreement on the need of cytogenetic examination at suspected diagnosis (43/43, 100%), agreement at progression (35/43, 81.4%), but disagreement on its use to assess response to HMA (9/43, 20.9%). Insufficient agreement was also found on the use of Fluorescent In-Situ Hybridization (FISH) panel (21/43, 48.8%) or array-Comparative Genomic Hybridization (array-CGH) in patients with normal karyotype (17/43, 39.5%). Agreement was found on performing molecular diagnostic (37/43, 86%) with comprehensive myeloid gene panels (29/37, 78.4%). In contrast, agreement was insufficient regarding the need for flow cytometry (28/43, 65.1%). While most participants were aware of the hereditary AML/MDS syndromes (33/43, 76.7%), there was insufficient agreement on the requirement to record a pedigree (23/33, 69.7%) and disagreement on using a systematic screening questionnaire (2/33, 6%) [25] as well as referring to genetic counseling (7/33, 21.2%) in suspicious cases of younger MDS patients.

### 3.2. Risk-Stratification

We received sufficient feedbacks for 10 of 12 questions (83%) in this domain, while questions about quality of life and geriatric assessment tools had to be dropped (Table 3 and Supplementary Materials Table S1). There was high agreement on applying risk-stratification with disease-based risk scores (40/42, 95.2%), whereas only about half of the participants (23/43, 54.8%) used patient-based risk scores. Most of the survey participants agreed on applying disease-based risk scores at diagnosis (41/42, 97.7%) and progression (31/42, 73.8%), while only about half agreed in using them at transplantation (24/42, 57.1%). The 23 participants, using patient-based risk-stratification, did it preferentially at diagnosis (78.2%) and transplantation (74%) but without a preference for a specific score. There was high agreement on using the *Revised International Prognostic Scoring System* (R-IPSS) (41/42, 97.6%) with insufficient agreement on the IPSS (28/42, 66.7%), the *WHO-prognostic Scoring System* (WPSS) (19/42, 45.2%) and the hypoplastic MDS prognostic risk score (18/39, 46.2%) for disease-based risk-stratification. Finally, we found disagreement on using tools for combined risk-stratification, for geriatric as well as quality of life (QoL) assessment.

### 3.3. Treatment

#### 3.3.1. Supportive Care

We received sufficient feedbacks for only eight of 21 questions (38%), while most of our questions on infectious prophylaxis had to be dropped (Table 4 and Supplementary Materials Table S1). There was insufficient agreement regarding the transfusion thresholds in all age groups regardless of comorbidities. Moreover, we found disagreement concerning the use of empirical primary infection prophylaxis (11/41, 26.8%) in patients with severe neutropenia (<0.5 g/L) and insufficient agreement on which compounds should be used, with exception of antimycotic agents (8/11, 72.7%).

#### 3.3.2. Growth Factors

We received sufficient feedbacks for 17 of 22 questions (77.3%), while questions on dosing of growth factors and neutrophil trigger for primary prophylaxis had to be dropped (Table 5 and Supplementary Materials Table S1). We observed agreement only on the use of darbepoetin alfa (31/42, 75.6%) but not on other recombinant erythropoiesis stimulating agents (ESA). There was insufficient agreement concerning target hemoglobin concentration, the time for dose adaptation, and the maximum weekly dose of recombinant ESA or darbepoietin alpha. We found disagreement on the use of granulocyte colony-stimulating factor (G-CSF) (10/41, 24.4%) or lenalidomide (LEN) (12/40, 30%) in ESA non-responders. There was clear disagreement on G-CSF for primary infection prophylaxis in neutropenic patients (4/41, 9.8%) but agreement on secondary prophylaxis in neutropenic patients after a history of fever (25/41, 61%), using a neutrophil trigger of <0.5 g/L (22/25, 88%). Insufficient agreement was found on the use of pegylated G-CSF (10/25, 40%). Concerning thrombopoietin-receptor agonists (TPO-RA), insufficient agreement was found on the type of agent, the threshold (tendency <20 G/L) and the appropriate indication in MDS patients (tendency for lower-risk MDS, concomitant ITP and hypoplastic MDS).

#### 3.3.3. Disease-Modifying Treatment

We received sufficient feedbacks for 28 of 40 questions (70%), while questions on thromboembolic prophylaxis with LEN, treatment modalities of immunosuppressive treatment (IST) and dose adjustments of HMA had to be dropped (Table 6 and Supplementary Materials Table S1). We found a high agreement on treatment of MDS del(5q) patients with LEN (39/40, 97.5%), agreement on treatment with LEN of patients with one or two additional cytogenetic alteration except chromosome 7 (29/38, 76.3%) and agreement on assessment of TP53 mutational status before LEN start (25/38, 65.8%) and in LEN non-responders (24/38, 63.2%). Insufficient agreement was found on previous assessment of ESA responsiveness (25/38, 65.8%) and the use of LEN in non-del(5q) MDS patients (13/39; 33.3%). The most frequent dosing schedule for LEN was 10 mg d1–d21 (65.8%). The participants disagreed on combining LEN with ESA (10/38, 26.3%) and using thromboembolic prophylaxis (11/39, 28.2%).

We found agreement on using anti-thymocyte globulin (ATG) with cyclosporin A (CsA) as IST in patients with hypoplastic MDS (28/39, 71.8%) but insufficient agreement on the first line treatment in younger patients (<40 years) (15/28, 53.6%). Disagreement was found on a strict combination of ATG/CsA in elderly patients (8/28, 28.6%) and strong disagreement on its use in MDS patients with other than hypoplastic MDS (2/39, 5%). There was insufficient agreement on the use of steroids in MDS patients (14/39, 35.9%) with potential indications comprising immune-thrombocytopenia and autoimmune hemolytic anaemia.

Concerning the requirements for initiation of treatment with HMA, we found agreement on an intermediate IPSS-R as minimal disease-based risk score (25/39, 64.1%), as well as the occurrence of severe thrombocytopenia (<20 G/L), anemia (<70 g/L) or neutropenia (<0.5 G/L) (25/39, 64.1%). The preferred HMA was azacytidine (38/39, 97.4%) prescribed most frequently at 100 mg/m^2^ d1–d5 (63.2%) compared to 75 mg/m^2^ d1–d7 (37.8%). Insufficient agreement was found on dose reduction in frail patients with non-high risk (17/39, 43.6%) and disagreement for dose reduction in high-risk MDS patients (9/39, 23.1%). We found agreement on performing bone-marrow assessment at the emergence of signs for progression (35/39, 89.7%), insufficient agreement on stable patients after 4–6 cycles (14/39, 35.9%) and disagreement on the documentation of best response (8/39, 20.5%). Disagreement was also found on dose reduction after six cycles in responding patients (1/39, 2.6%) or extension of the treatment intervals (9/39, 23.1%), both of which might be reserved for patients with side effects.

#### 3.3.4. Allogeneic Hematopoietic Stem Cell Transplantation

Only six participants declared experience with allo HSCT, which reduced the representativeness of our survey for this treatment option. We received more than 50% feedbacks for 14 of 22 questions (63.6%) (Table 7 and Supplementary Materials Table S1). 

There was agreement on the maximum age for transplantation (70–74 years), the consideration of patients with only high-risk disease (IPSS intermediate 2 and IPSS-R high, very high) and high-risk molecular risk factors for the indication of allo HSCT (all 5/6, 83.3%). Insufficient agreement was found on the question, if intermediate fit (2/6, 33.3%) or only unfit patients (4/6, 66.7%) (according HCT-CI and MDS-CI) should be precluded from allo HSCT. Furthermore, there was agreement on the treatment with standard induction chemotherapy for fit patients with excess of blast (5/6, 83.3%) and full agreement on the use of reduced-intensity conditioning regimen (RIC) for intermediate fit patients with <5% BM blasts before transplantation (6/6, 100%). We found a high disagreement on performing autologous HSCT as consolidation treatment for MDS patients without a suitable donor (0/6, 0%).

## 4. Discussion

With our survey, we have captured the current perception among hemato-oncologists of what they consider a recommended management of adult MDS patients in Switzerland. Here we mainly focused on areas with uncertain agreement, which can be addressed for future improvements.

In the diagnostic domain, the participants expressed their uncertainty in using the diagnostic terms of CHIP, CCUS, ICUS/IDUS. This probably reflects insufficient familiarity with these recent terms, their questionable relevance in daily clinical practice and issues with reimbursement of NGS in patients with mild cytopenia. The uncertainty in using bone marrow examination and cytogenetic to assess response to HMA is most likely due to limited treatment alternatives after HMA failure in elderly patients. Moreover, the role of flowcytometry for diagnosis and prognosis in MDS remains controversial, not only in our survey but also among international MDS experts [36]. Flowcytometry requires a high degree of infrastructural and personnel resources, standardization and might be replaced by NGS for the identification of clonality in uncertain cases. Recording a pedigree, using screening questionnaires and referral for genetic counseling seem to be reasonable in patients with suspected germ-line predisposition but did not reach sufficient agreement in our survey, similarly as we have observed among international MDS experts [36]. However, due to the simplicity of an adequate history taking and significant consequences for allo HSCT (donor selection and conditioning intensity), we would like to emphasize the importance of correctly diagnosing familial cases of MDS.

In the risk-stratification domain, disease-based risk-stratification with IPSS did not reach sufficient agreement, in contrast to IPSS-R. This seems to be surprising, as IPSS is required for appropriate treatment allocation according to official labels derived from remote clinical trials, even though, IPSS-R performs much better for risk-stratification [13]. Therefore, both seem to have their justification. There is a broad uncertainty in using patient-based risk scores, combined risk-stratification, geriatric and quality of life (QoL) assessment, which is in line with our observation made with international MDS experts [36]. Even though, these are relevant aspects for the management of elderly MDS patients, there is an unmet need in finding a consensus for their appropriate use in daily clinical practice.

Regarding the therapy domain, most questions on infectious prophylaxis received insufficient feedbacks and participants expressed disagreement concerning the use of empirical primary infection prophylaxis. This may reflect the lack of available evidence from appropriately designed clinical trials leading to insufficient consensus on this topic. In addition, due to concerns of bacterial resistance there is a general reluctance in using antibiotic prophylaxis. Regarding treatment with ESAs in lower-risk MDS patients, we found agreement on the use of darbepoetin alfa but not for other recombinant ESAs. This might be due to the fact that darbepoietin alpha is the only ESA reimbursed for the MDS indication in Switzerland and needs applications only every 2–3 weeks [37]. However, there is no formal proof of its superiority in efficacy or safety compared to other ESAs. We also found uncertainty concerning target hemoglobin level, dosing schedules and dose adjustments in our survey, which might reflect insufficient standardization of ESA treatment and assessment of response. Concerning TPO-RA, we found insufficient agreement for the appropriate indication in MDS, the preferred agent and the trigger of platelet counts required for initiation of treatment. While TPO-RA have shown to increase platelet counts, reduce the number of platelet transfusions and severity of bleeding episodes in randomized controlled trials [38,39], the overall clinical benefit remains unclear and the treatment is still off-label. Furthermore, initial concerns of potential disease progression and leukemic transformation seem not to be justified but remain potential reasons for reluctance [40]. We found low feedbacks on thromboembolic prophylaxis with LEN, treatment modalities of IST and dosing adjustments of HMA, which may be explained by insufficient data from clinical trials. Furthermore, the insufficient agreement on the first line treatment in younger patients (<40 years) and disagreement for strict combination of ATG/CsA in elderly patients with hypoplastic MDS most likely reflects the lack of experience and standardized approaches with this rare MDS entity. The low feedback rate regarding allo HSCT from only six knowledgeable participants limits the interpretation of these results. We observed insufficient agreement for the decision, if intermediate fit or only unfit patients (according HCT-CI and MDS-CI) should be precluded from allo HSCT, which underlines the lack of evidence for this question. The fact that none of the participants gave any preference for the induction or consolidation treatment regimen before allo HSCT reflects previous observations we made during the GBI development [36].

The strengths of our study was the wide range of questions, covering many aspects of clinical care of MDS patients. Moreover, the participants were affiliated to different types of health care centers and the determination of agreement/disagreement was standardized. The main limitation is the low number of participants (overall feedback-rate 14%), which is, however, inherent to web-based surveys among physicians [41]. Our survey reflects a national perspective of physicians that are interested in MDS treatment, who were mainly hematologists <50 years of age, associated to the Swiss hematology association and practicing in secondary and tertiary centers with few participants from primary centers. This may limit the extrapolation to all of Switzerland and other countries. It is also known form other studies that tertiary centers treat younger patients with more intensive treatments, which translated into a reduced mortality in academic compared to community-based hospitals [42] Moreover, the survey was not designed to provide a feedback to the participants, discuss the results and repeat the assessment. This is usually done in a more structured RAND DELPHI consensus finding process, as we have previously adopted for the GBI development [36,43] As such, surveys remain generally biased by the limited number and the composition of the participants. Finally, and most importantly, agreeing or dis-agreeing with certain questions does not necessarily mean that the clinician’s attitude will be translated into practice.

The main reasons why clinicians may not follow G/Rs include awareness, familiarity, agreement with the contents, insufficient evidence with questionable clinical relevance but also potential issues regarding reimbursement [44]. There are also potential patient-based factors that interfere with recommended management such as advanced comorbidities and frailty, acceptance of treatment and compliance. Moreover, also provider and infrastructural barriers may influence adherence, such as limited time and personnel (physicians, nurses, multidisciplinary care teams), inadequate organization (isolation ward, handling of cytotoxic drugs, interdisciplinary boards, emergency services, standard operating procedures), barriers for cooperation (exchange of knowledge, multi-institutional care networks, access to clinical trials) as well as financial resources. However, reasons for adherence/non-adherence and their impact on relevant patient centered outcomes remain obscure and have not been sufficiently investigated. This may be caused by the fact, that process-based elements for quality of care and patient centred outcomes are not systematically measured in daily clinical routine [45,46]. For this, quality indicators need to be defined as “measurable elements of practice performance for which there is evidence or consensus that they can be used to assess and change the quality of care provided” [45]. Quality indicators are usually extracted from published, evidence-based guidelines by a structured selection and consensus process, and are referred to as guideline-based indicators (GBIs). Motivated by all these relevant limitations, our study group set out to develop a first consensus on GBIs, addressing best practice performance, outcomes and structural resources in collaboration with internationally acknowledged experts [36,47,48]. Such GBIs have been recently published for adult MDS patients and will be prospectively validated as standardized instrument in the I-CARE for MDS Study with the goal to assess, compare and foster good quality of care.

## 5. Conclusions

We performed a survey in hemato-oncologists focusing on recommended care in adult MDS patients in Switzerland. We identified areas for improvement in the domains of diagnosis, risk-stratification and therapy, which should be further addressed with clinical trials, information, and guiding documents. Identifying reasons for adherence/non-adherence to G/Rs is important to understand the clinical challenges for their implementation, areas with insufficient evidence and potential fields of action. A standardized assessment of relevant GBIs may help to define specific measures to improve daily care of MDS patients within clinical development cycles.

## Figures and Tables

**Table 1:** Characteristics of participants.

Total		43 (100%)
Age-categories		
	30–40 years	10 (23.2%)
	41–50 years	20 (46.5%)
	51–60 years	9 (20.9%)
	61–70 years	2 (4.7%)
	Missing	2 (4.7%)
Year of board certification		
	1986–2000	7 (16.3%)
	2001–2010	13 (30.2%)
	2011–2017	16 (37.2%)
	Missing	7 (16.3%)
Institution		
	General practice	1 (2.3%)
	Specialised practice	5 (11.6%)
	Primary center	5 (11.6%)
	Secondary center	14 (32.6%)
	Tertiary center	18 (41.9%)
	Missing	-
Medical association membership		
	SSH	36 (90%)
	EHA	25 (62.5%)
	ASH	13 (30%)
	SMSG	7 (17.5%)
	DGHO	7 (17.5%)
	SSMO	4 (10%)
	Missing	3 (7%)
Agreement with ELN 2013 recommendations for diagnosis of MDS(1)	Yes	38 (88.3%)
	No	2 (4.7%)
	Not familiar	3 (7%)
Agreement with ELN 2013 recommendations for treatment of MDS(1)	Yes	37 (86%)
	No	2 (4.7%)
	Not familiar	3 (7%)
	Missing	1 (2.3%)

ASH: American Society of Hematology, DGHO: German Society of Hematology and Medical Oncology, EHA: European Hematology Association, SSH: Swiss Society of Hematology, SSMO: Swiss Society of Medical Oncology, SMSG: Swiss MDS Study Group.

**Table 2:** Diagnostic evaluation.

Questions	Feedback	Performance
**1. Classification**		
MDS classification system	43 (100%)	WHO 2016 ([23]): 41 (95.3%) WHO 2008 ([26]): 12 (27.9%) FAB ([27]): 3 (7%)
Use of the diagnostic terms ICUS, IDUS, CCUS or CHIP [24]	43 (100%)	Yes: 29 (67.4%) No: 14 (32.6%)
**2. Bone Marrow (BM)**		
Time of the BM assessment	43 (100%)	At (suspected) diagnosis: 43 (100.0%) At (suspected) progression: 40 (93.0%) To access response to hypomethylating agents (HMAs): 20 (46.5%)
Who evaluates BM smears at your institution?	43 (100%)	Hematologist: 41 (95.3%) Pathologist: 10 (23.3%)
Acceptable inter-observer variability of morphological signs of dysplasia	43 (100%)	Yes: 24 (55.8%) No: 19 (44.2%)
Does morphological quantification of blasts have an acceptable inter-observer variability?	43 (100%)	Yes: 31 (72.1%) No: 12 (27.9%)
**3. Cytogenetics/FISH**		
When should karyotyping be performed?	43 (100%)	At (suspected) diagnosis: 43 (100.0%) At (suspected) progression: 35 (81.4%) To assess response to hypomethylating agents (HMAs): 9 (20.9%)
Necessity of analysis with an MDS FISH panel in MDS patients with normal karyotype	43 (100%)	Yes: 21 (48.8%) No: 15 (34.9%) Undecided: 7 (16.3%)
Should array-CGH replace MDS FISH panels in MDS patients with normal karyotype?	43 (100%)	Yes: 17 (39.5%) Undecided: 14 (32.6%) No: 12 (27.9%)
**4. Molecular Diagnostics**		
Do you use molecular diagnostics for MDS?	43 (100%)	Yes: 37 (86.0%) No: 6 (14.0%)
What type of gene panel do you use for diagnosis?	37 (86%)	Comprehensive myeloid driver gene panel (>10 genes): 29 (78.4%) Undecided: 5 (13.5%) Only single gene analysis: 3 (8.1%) Reduced myeloid driver gene panel: 0 (0.0%)
**5. Flow Cytometry**		
Do you use flow cytometry for MDS diagnosis?	43 (100%)	Yes: 28 (65.1%) No: 15 (34.9%)
Do you use the OGATA flow cytometry score for MDS diagnosis [28]?	28 (65.1%)	Yes: 18 (64.3%) I am not familiar with OGATA flow score: 5 (17.9%) No: 4 (14.3%) I am using another flow score: 1 (3.6%)
**6. Germ Line Predisposition**		
Are you aware of hereditary AML/MDS syndromes?	43 (100%)	Yes: 33 (76.7%) No: 10 (23.3%)
Do you record a pedigree for patients with suspected hereditary AML/MDS syndromes?	33 (76.7%)	Yes: 23 (69.7%) No: 10 (30.3%)
Do you use a questionnaire for the screening of patients with suspected hereditary AML/MDS syndromes [25]?	33 (76.7%)	No: 31 (93.9%) Yes: 2 (6.1%)
Have you ever referred a patient to genetic counseling due to suspected hereditary AML/MDS syndrome?	33 (76.7%)	No: 26 (78.8%) Yes: 7 (21.2%)

**Table 3:** Risk-stratification.

Questions	Feedback	Performance
Should assessment of disease- and patient-based risk factors be performed?	42 (97.7%)	Yes: 40 (95.2%) Undecided: 2 (4.8%) No: 0 (0.0%)
**1. Disease-Based Risk-Stratification**		
When do you use disease-based risk-stratification scores in MDS patients?	42 (97.7%)	At diagnosis: 41 (97.6%) At progression: 31 (73.8%) At transplantation: 24 (57.1%) Other time points: 1 (2.4%) Not at all: 0 (0.0%)
Which of the following scores do you use for disease-based risk-stratification?	42 (97.7%)	R-IPSS/IPSS-R [29]: 41 (97.6%) IPSS [30]: 28 (66.7%) WPSS [31]: 19 (45.2%) Other: 0 (0.0%)
**2. Patient-Based Risk-Stratification**		
Do you use patient-based risk-stratification scores in all MDS patients?	42 (97.7%)	Yes: 23 (54.8%) No: 19 (45.2%)
When do you use patient-based risk-stratification scores in MDS patients?	23 (53.5%)	At diagnosis: 18 (78.3%) At transplantation: 17 (73.9%) At progression: 13 (56.5%) Other time points: 1 (4.3%) Not at all: 1 (4.3%)
Which of the following scores do you use for patient-based risk-stratification?	39 (90.7%)	HCT-CI (Hematopoietic Cell Transplantation-specific comorbidity index) [32]: 26 (66.7%) MDS-CI (MDS comorbidity index) [33]: 14 (35.9%) CCI (Charlson comorbidity index) [34]: 12 (30.8%) Other: 5 (12.8%)
**3. Others**		
Do you use the prognostic model for hypoplastic MDS [35]?	39 (90.7%)	I am not familiar: 15 (38.5%) Yes: 13 (33.3%) No: 11 (28.2%)
Do you use a combined risk-stratification tool?	42 (97.7%)	No: 39 (92.9%) Yes: 3 (7.1%)
Do you use geriatric assessment tools to decide, if elderly high-risk MDS patients with excess of blasts are eligible for standard induction chemotherapy?	42 (97.7%)	No: 30 (71.4%) Yes: 12 (28.6%)
Do you use quality of life (QoL) assessment tools?	42 (97.7%)	No: 37 (88.1%) Yes: 5 (11.9%)

**Table 4:** Supportive care.

Questions	Feedback	Performance
**1. RBC and TC Transfusions**		
Hemoglobin threshold for RBC transfusion for younger patients (<70 years) without comorbidities?	41 (95.3%)	Only if symptomatic: 4 (9.8%) < 60 g/L: 10 (24.4%) < 70 g/L: 17 (41.5%) < 80 g/L 8 (19.5%) < 90 g/L: 1 (2.4%) < 100 g/L: 0 (0.0%) Other: 1 (2.4%)
Hemoglobin threshold for RBC transfusion for younger patients (<70 years) with relevant cardiovascular or pulmonary comorbidities?	41 (95.3%)	Only if symptomatic: 2 (4.9%) < 60 g/L: 0 (0.0%) < 70 g/L: 13 (31.7%) < 80 g/L: 19 (46.3%) < 90 g/L: 4 (9.8%) < 100 g/L: 1 (2.4%) Other: 2 (4.9%)
Hemoglobin threshold for RBC transfusion for elderly patients (≥70 years) without comorbidities?	41 (95.3%)	Only if symptomatic: 3 (7.3%) < 60 g/L: 0 (0.0%) < 70 g/L: 16 (39.0%) < 80 g/L: 18 (43.9%) < 90 g/L: 3 (7.3%) < 100 g/L: 0 (0.0%) Other: 1 (2.4%)
Hemoglobin threshold for RBC transfusion for elderly patients (≥70 years) with relevant cardiovascular or pulmonary comorbidities?	41 (95.3%)	Only if symptomatic: 1 (2.4%) < 60 g/L: 0 (0.0%) < 70 g/L: 2 (4.9%) < 80 g/L: 16 (39.0%) < 90 g/L: 14 (34.1%) < 100 g/L: 6 (14.6%) Other: 2 (4.9%)
Platelet threshold for transfusion for younger patients (<70 years) without signs of bleeding or infection?	41 (95.3%)	Only in case of clinically relevant bleeding: 8 (19.5%) < 5 G/L: 8 (19.5%) < 10 G/L: 24 (58.5%) < 20 G/L: 0 (0.0%) < 30 G/L: 0 (0.0%) Other: 1 (2.4%)
Platelet threshold for transfusion for elderly patients (≥70 years) without signs of bleeding or infection?	41 (95.3%)	Only in case of clinically relevant bleeding: 7 (17.1%) < 5 G/L: 8 (19.5%) < 10 G/L: 25 (61.0%) < 20 G/L: 1 (2.4%) < 30 G/L: 0 (0.0%) Other: 0 (0.0%)
Platelet threshold for patients with signs of infection?	41 (95.3%)	Only in case of clinically relevant bleeding: 1 (2.4%) < 5 G/L: 0 (0.0%) < 10 G/L: 20 (48.8%) < 20 G/L: 19 (46.3%) < 30 G/L: 1 (2.4%) Other: 0 (0.0%)
**2. Infection Prophylaxis**		
Do you provide empirical infection prophylaxis in patients with severe neutropenia (<0.5 G/L)?	41 (95.3%)	No: 26 (63.4%) Yes: 11 (26.8%) Only after previous infection: 4 (9.8%)

**Table 5:** Growth factors.

Questions	Feedback	Performance
**1. Erythropoietin Stimulating Agents (ESA)**		
Do you agree with the ELN 2013 recommendations for ESA treatment in MDS patients [1]?	41 (95.3%)	Yes: 34 (82.9%) I am not familiar: 4 (9.8%) No: 3 (7.3%)
Which ESA do you prefer?	41 (95.3%)	Darbepoetin alfa (Aranesp^®^): 31 (75.6%) No preference: 5 (12.2%) Epoetin alfa (e.g., Eprex^®^): 3 (7.3%) Epoetin beta (e.g., Recormon^®^): 2 (4.9%)
What is the Hemoglobin concentration you aim for (g/L)?	41 (95.3%)	80–89: 6 (14.6%) 90–99: 7 (17.1%) 100–109: 21 (51.2%) 110–119: 7 (17.1%) ≥120: 0 (0.0%)
After what time do you adapt the initial dose (weeks)?	40 (93%)	0: 2 (5%) 2: 5 (12.5%) 4: 15 (37.5%) 6: 10 (25%) 8: 7 (17.5%) 10: 0 (0.0%) 12: 1 (2.5%)
What is the maximum dose you use in order to test ESA responsiveness for epoetin beta?	15 (total response: 35, 81.4%)	10,000: 1 (6.6%) 30,000: 4 (26.7%) 40,000: 1 (6.6%) 60,000: 4 (26.7%) 80,000: 4 (26.8%) 100,000: 1 (6.6%)
What is the maximum dose you use in order to test ESA responsiveness for darbepoetin alfa?	20 (total response: 35, 81.4%)	150: 1 (5%) 250: 1 (5%) 300: 2 (10%) 500: 16 (80%)
Do you add G-CSF in non-responders?	41 (95.3%)	No: 31 (75.6%) Yes: 10 (24.4%)
Do you try lenalidomide in non-responders?	40 (93%)	Only in del(5q): 22 (55%) Yes: 12 (30%) No: 6 (15%)
**2. Granulocyte-Colony-Stimulating Factor (G-CSF)**		
Do you use G-CSF for primary infection prophylaxis in neutropenic patients?	41 (95.3%)	No: 37 (90.2%) Yes: 4 (9.8%)
Do you use G-CSF for secondary infection prophylaxis in neutropenic patients that experienced neutropenic fever?	41 (95.3%)	Yes: 25 (61%) No: 16 (39%)
What is your neutrophil trigger for secondary infection prophylaxis?	25 (58.1%)	<1.5 G/L: 0 (0.0%) <1.0 G/L: 2 (8%) <0.5 G/L: 22 (88%) <0.2 G/L: 1 (4%)
Do you use pegylated G-CSF?	26 (60.5%)	No: 16 (61.5%) Yes: 10 (38.5%)
**3. Thrombopoietin-Receptor Agonist (TPO-RA)**		
Do you use TPO-RA in thrombopenic MDS patients? (multiple answers possible)	40 (93%)	In MDS with concomitant ITP: 18 (45%) In hypoplastic MDS: 15 (37.5%) Not at all: 13 (32.5%) In lower-risk MDS (IPSS low, int-1): 12 (30%) As secondary bleeding prophylaxis: 6 (15%) In higher-risk MDS (IPSS int-2, high): 2 (5%) As primary bleeding prophylaxis: 1 (2.5%)
Which TPO-RA do you prefer?	26 (60.5%)	I do not have a preference: 11 (42.3%) Romiplostim (Nplate^®^): 8 (30.8%) Eltrombopag (Revolade^®^): 7 (26.9%)
Which platelet count trigger do you use for secondary TPO-RA treatment (G/L)?	27 (62.8%)	20: 11 (40.7%) 10: 9 (33.3%) 30: 6 (22.2%) 5: 1 (3.7%) 50: 0 (0.0%)
Which maximum dose do you use for eltrombopag?	18 (total response: 27, 62.8%)	100 mg (50 mg asian)/day: 7 (25.9%) 200 mg (100 mg asian)/day: 6 (22.2%) 300 mg (150 mg asian)/day: 5 (18.5%)
Which maximum dose do you use for romiplostim?	19 (total response: 27, 62.8%)	10 ug/kg/week: 14 (51.9%) 5 ug/kg/week: 2 (7.4%) Other: 3 (11.1%)

**Table 6:** Disease-modifying treatment.

Questions	Feedback	Performance
**1. Lenalidomid (LEN)**		
Do you use LEN in del(5q) MDS patients?	40 (93%)	Yes: 39 (97.5%) No: 1 (2.5%)
Which initial LEN dose do you use in MDS patients?	38 (88.4%)	10 mg d1–21: 26 (68.4%) 10 mg d1–28: 7 (18.4%) 5 mg d1–21: 2 (5.3%) 5 mg d1–28: 2 (5.3%) Other dose: 1 (2.6%)
Do you assess EPO responsiveness before LEN?	38 (88.4%)	Yes: 25 (65.8%) No: 13 (34.2%)
Do you combine LEN and EPO?	38 (88.4%)	No: 28 (73.7%) Yes: 10 (26.3%)
Do you use LEN in del(5q) with additional cytogenetic alterations (except alterations of chromosome 7)?	38 (88.4%)	Yes: 29 (76.3%) No: 9 (23.7%)
How many cytogenetic alterations would you accept beside del(5q) to treat with LEN?	29 (67.4%)	1 additional alteration: 12 (41.4%) 2 additional alterations: 8 (27.6%) 3 additional alterations: 2 (6.9%) More than 3 additional alterations: 7 (24.1%)
Do you check TP53 mutational status before prescribing LEN?	38 (88.4%)	Yes: 25 (65.8%) No: 13 (34.2%)
Do you check TP53 mutational status in non-responders to LEN?	38 (88.4%)	Yes: 24 (63.2%) No: 14 (36.8%)
Do you use LEN in non-del(5q) MDS patients?	39 (90.7%)	No: 26 (66.7%) Yes: 13 (33.3%)
Do you provide thromboembolic prophylaxis when prescribing LEN?	39 (90.7%)	No: 28 (71.8%) Yes: 11 (28.2%)
**2. Immunosuppressive Treatment (IST)**		
Do you treat patient with hypoplastic MDS with ATG/CsA?	39 (90.7%)	Yes: 28 (71.8%) No: 11 (28.2%)
Do you try ATG/CsA in MDS patients with other than hypoplastic MDS?	39 (90.7%)	No: 37 (94.9%) Yes: 2 (5.1%)
Is ATG/CsA your first choice in younger patients (<40 years) with hypoplastic MDS?	28 (65.1%)	Yes: 15 (53.6%) No: 13 (46.4%)
Do you always combine CsA with ATG in elderly patients (>70 years) with hypoplastic MDS?	28 (65.1%)	No: 20 (71.4%) Yes: 8 (28.6%)
Do you use steroids in MDS patients?	39 (90.7%)	No: 25 (64.1%) Yes: 14 (35.9%)
**3. Hypomethylating Agents (HMA)**		
What minimal risk category do you require for treatment with HMA?	39 (90.7%)	Intermediate (IPSS: Int-1; IPSS-R: Intermediate): 25 (64.1%) High (IPSS: Int-2, high; IPSS-R: high, very high): 11 (28.2%) IPSS score does not influence my decision for HMA: 3 (7.7%) Low (IPSS: Low; IPSS-R: Very Low & Low): 0 (0.0%)
Are severe thrombopenia (<20 G/L), anemia (<70 g/L) or neutropenia (<0.5 G/L) an indication for treatment irrespective of IPSS or IPSS-R?	39 (90.7%)	Yes: 25 (64.1%) No: 14 (35.9%)
Which HMA do you preferably use for MDS patients?	39 (90.7%)	5-Azacytidine (AZA, Vidaza^®^): 38 (97.4%) Decitabine (DEC, Dacogen^®^): 1 (2.6%)
What is the standard dose you use for 5-Azacytidine (AZA)?	38 (88.4%)	100 mg/m^2^ d1–d5: 24 (63.2%) 75 mg/m^2^ d1–d7: 14 (36.8%) Other: 0 (0.0%)
Do you reduce the dose in frail patients with non-high-risk MDS?	39 (90.7%)	No: 22 (56.4%) Yes: 17 (43.6%)
Do you reduce the dose in frail patients with high-risk MDS?	39 (90.7%)	No: 30 (76.9%) Yes: 9 (23.1%)
To which extent do you reduce the dose of HMA in frail MDS patients?	37 (86%)	75% of standard dose: 14 (37.8%) 50% of standard dose: 6 (16.2%) 25% of standard dose: 6 (16.2%) Other dose reduction: 11 (29.7%)
Do you perform BM aspiration in MDS patients under HMA therapy at best response?	39 (90.7%)	Not on a regular basis: 23 (59%) Yes: 8 (20.5%) No: 8 (20.5%)
Do you perform BM aspiration in MDS patients under HMA therapy in stable patients after 4–6 cycles?	39 (90.7%)	Not on a regular basis: 19 (48.7%) Yes: 14 (35.9%) No: 6 (15.4%)
Do you perform BM aspiration in MDS patients under HMA therapy at progression?	39 (90.7%)	Yes: 35 (89.7%) Not on a regular basis: 4 (10.3%) No: 0 (0.0%)
Do you reduce the dose of HMA in responding patients after 6 cycles?	39 (90.7%)	No: 24 (61.5%) Only in patients with side effects: 16 (41%) Yes: 1 (2.6%)
Do you extend the interval of HMA application in responding patients?	39 (90.7%)	No: 17 (43.6%) Yes: 9 (23.1%) Only in patients with side effects: 13 (33.3%)
Do you think that stable disease after 6 cycles in high-risk MDS patients can be considered as response?	39 (90.7%)	Yes: 26 (66.7%) No: 13 (33.3%)

**Table 7:** Allogeneic hematopoietic stem cell transplantation (*n* = 6).

Questions	Feedback	Performance
Do you perform allo HSCT at your center?	40 (93%)	Yes: 6 (15%) No: 34 (85%)
**1. Indications**		
At what risk category do you envisage allo HSCT?	6 (100%)	High (IPSS: Int-2, high; IPSS-R: high, very high): 5 (83.3%) Intermediate (IPSS: Int-1; IPSS-R: Intermediate): 1 (16.7%) Low (IPSS: Low; IPSS-R: Very Low & Low): 0 (0.0%) IPSS score does not influence my decision for allo HSCT: 0 (0.0%)
What is the maximum age to envisage allo HSCT?	6 (100%)	65–69 years: 1 (16.7%) 70–74 years: 5 (83.3%) >75 years: 0 (0.0%)
Which HCT-CI/MDS-CI score precludes allo HSCT?	6 (100%)	HCT-CI/MDS-CI ≥3 (less fit): 4 (66.7%) HCT-CI/MDS-CI 1-2 (intermediate fit): 2 (33.3%) HCT-CI/MDS-CI 0 (fit): 0 (0.0%)
**2. Induction Therapy**		
What is your preferred induction therapy for fit MDS patients (HCT-CI/MDS-CI: 0) with <5% BM-blasts eligible for allo HSCT?	6 (100%)	HMA: 3 (50.0%) No induction, upfront allo HSCT: 2 (33.3%) Standard AML induction chemotherapy (e.g., 3 + 7): 1 (16.7%) Other: 0 (0.0%)
What is your preferred induction therapy for fit MDS patients (HCT-CI/MDS-CI: 0) with >5% BM-blasts eligible for allo HSCT?	6 (100%)	Standard AML induction chemotherapy (e.g., 3 + 7): 5 (83.3%) HMA: 1 (16.7%) No induction, upfront allo HSCT: 0 (0.0%) Other: 0 (0.0%)
What is your preferred induction therapy for intermediate fit MDS patients (HCT-CI/MDS-CI: 1–2) with <5% BM-blasts eligible for allo HSCT?	6 (100%)	HMA: 3 (50.0%) No induction, upfront allo HSCT: 3 (50.0%) Standard AML induction chemotherapy (e.g., 3 + 7): 0 (0.0%) Other: 0 (0.0%)
What is your preferred induction therapy for intermediate fit MDS patients (HCT-CI/MDS-CI: 1-2) with >5% BM-blasts eligible for allo HSCT?	6 (100%)	HMA: 4 (66.7%) Standard AML induction chemotherapy (e.g., 3 + 7): 2 (33.3%) No induction, upfront allo HSCT: 0 (0.0%) Other: 0 (0.0%)
**3. Conditioning Therapy**		
What is your preferred conditioning strategy for fit MDS patients (HCT-CI/MDS-CI: 0) with <5% BM-blasts eligible for allo HSCT?	6 (100%)	RIC: 3 (50.0%) MAC: 3 (50.0%)
What is your preferred conditioning strategy for fit MDS patients (HCT-CI/MDS-CI: 0) with >5% BM-blasts eligible for allo HSCT?	6 (100%)	RIC: 3 (50.0%) MAC: 3 (50.0%)
What is your preferred conditioning strategy for intermediate fit MDS patients (HCT-CI/MDS-CI: 1–2) with <5% BM-blasts eligible for allo HSCT?	6 (100%)	RIC: 6 (100.0%) MAC: 0 (0.0%)
What is your preferred conditioning strategy for intermediate fit MDS patients (HCT-CI/MDS-CI: 1–2) with >5% BM-blasts eligible for allo HSCT?	6 (100%)	RIC: 5 (83.3%) MAC: 1 (16.7%)
**4. Others**		
Do you perform autologous HSCT as a consolidation therapy for MDS patients without a suitable donor?	6 (100%)	No: 6 (100%) Yes: 0 (0.0%)
Do you consider molecular risk factors such as TP53 or RAS for the decision to transplant or not?	6 (100%)	Yes: 5 (83.3%) No: 1 (16.7%)

## Supplementary Materials

The following are available online at https://www.mdpi.com/1660-4601/17/24/9562/s1. Table S1: Questions with insufficient feedbacks.

## Abbreviations

ATG	Anti-thymocyte globulin
AML	Acute myeloid leukemia
BM	Bone marrow
CCUS	Clonal cytopenia of unknown significance
CGH	Comparative Genomic Hybridization
CHIP	Clonal hematopoiesis of indeterminate potential
COS	Core outcome set
CsA	Cyclosporin A
DGHO	Deutsche Gesellschaft für Hämatologie und Onkologie
DKG	Deutsche Krebs Gesellschaft
EHA	European Hematology Association
ELN	European Leukemia Net
EPO	Erythropoietin
ESA	Erythropoietin Stimulating Agents
ESMO	European Society of Medical Oncology
FISH	Fluorescent In-Situ Hybridisation
GA	Geriatric assessment
G-CSF	Granulocyte Colony-Stimulating Factor
G/Rs	Guidelines and recommendations
HCT-CI	Hematopoietic Cell Transplantation Comorbidity Index
HI	Hematological improvement
HMA	Hypomethylating agents
HSCT	Hematopoietic stem cell transplantation
ICUS	Idiopathic cytopenia of unknown significance
IDUS	Idiopathic dysplasia of unknown significance
IP	Immunophenotyping
IPSS	International Prognostic Scoring System
IPSS-R	Revised International Prognostic Scoring System
IST	Immunosuppressive Treatment
LEN	Lenalidomide
MDS	Myelodysplastic syndromes
PB	Peripheral blood
SMSG	Swiss MDS Study Group
SSH	Swiss Society of Hematology
TPO-RA	Thrombopoietin receptor agonists
QoL	Quality of life
WHO	World Health Organisation
WPSS	WHO Prognostic Scoring System

## References

[1] Malcovati L., Hellstrom-Lindberg E., Bowen D., Ades L., Cermak J., Del Canizo C., Della Porta M.G., Fenaux P., Gattermann N., Germing U. (2013). Diagnosis and treatment of primary myelodysplastic syndromes in adults: Recommendations from the European LeukemiaNet. Blood.

[2] https://www.nccn.org/professionals/physician_gls/default.aspx#site.

[3] Fenaux P., Haase D., Sanz G.F., Santini V., Buske C., Group E.G.W. (2014). Myelodysplastic syndromes: ESMO Clinical Practice Guidelines for diagnosis, treatment and follow-up. Ann. Oncol..

[4] Bonadies N., Feller A., Rovo A., Ruefer A., Blum S., Gerber B., Stuessi G., Benz R., Cantoni N., Holbro A. (2017). Trends of classification, incidence, mortality, and survival of MDS patients in Switzerland between 2001 and 2012. Cancer Epidemiol..

[5] Roman E., Smith A., Appleton S., Crouch S., Kelly R., Kinsey S., Cargo C., Patmore R. (2016). Myeloid malignancies in the real-world: Occurrence, progression and survival in the UK’s population-based Haematological Malignancy Research Network 2004-15. Cancer Epidemiol..

[6] Campbell S.M., Roland M.O., Buetow S.A. (2000). Defining quality of care. Soc. Sci. Med..

[7] Grimshaw J.M., Russell I.T. (1993). Effect of clinical guidelines on medical practice: A systematic review of rigorous evaluations. Lancet.

[8] Bero L.A., Grilli R., Grimshaw J.M., Harvey E., Oxman A.D., Thomson M.A. (1998). Closing the gap between research and practice: An overview of systematic reviews of interventions to promote the implementation of research findings. The Cochrane Effective Practice and Organization of Care Review Group. BMJ.

[9] Graham I.D., Stiell I.G., Laupacis A., McAuley L., Howell M., Clancy M., Durieux P., Simon N., Emparanza J.I., Aginaga J.R. (2001). Awareness and use of the Ottawa ankle and knee rules in 5 countries: Can publication alone be enough to change practice?. Ann. Emerg. Med..

[10] Lomas J., Anderson G.M., Domnick-Pierre K., Vayda E., Enkin M.W., Hannah W.J. (1989). Do practice guidelines guide practice? The effect of a consensus statement on the practice of physicians. N. Engl. J. Med..

[11] Porter A., Iakobishvili Z., Dictiar R., Behar S., Hod H., Gottlieb S., Hammerman H., Zahger D., Hasdai D. (2007). The implementation of guidelines and prognosis among patients with acute coronary syndromes is influenced by physicians’ perception of antecedent physical and cognitive status. Cardiology.

[12] Abel G.A., Cronin A.M., Odejide O.O., Uno H., Stone R.M., Steensma D.P. (2016). Influence of patient and provider characteristics on quality of care for the myelodysplastic syndromes. Br. J. Haematol..

[13] Moreno Berggren D., Folkvaljon Y., Engvall M., Sundberg J., Lambe M., Antunovic P., Garelius H., Lorenz F., Nilsson L., Rasmussen B. (2018). Prognostic scoring systems for myelodysplastic syndromes (MDS) in a population-based setting: A report from the Swedish MDS register. Br. J. Haematol..

[14] Gattermann N., Kundgen A., Kellermann L., Zeffel M., Paessens B., Germing U. (2013). The impact of age on the diagnosis and therapy of myelodysplastic syndromes: Results from a retrospective multicenter analysis in Germany. Eur. J. Haematol..

[15] Santini D., Della Porta M., Enrico B., Pelizzari A., Molteni A., Riva M., Poloni A., Musto P., Finelli C., Ferrero D. Evaluation of Adherence to Treatment Recommendations According to Italian and European Guidleines in MDS Patients Enrolled in the Italian FISiM Registry. Proceedings of the 15th International Symposium on Myelodysplastic Syndromes.

[16] Dinmohamed A.G., van Norden Y., Visser O., Posthuma E.F., Huijgens P.C., Sonneveld P., van de Loosdrecht A.A., Jongen-Lavrencic M. (2015). The use of medical claims to assess incidence, diagnostic procedures and initial treatment of myelodysplastic syndromes and chronic myelomonocytic leukemia in the Netherlands. Leuk. Res..

[17] Dinmohamed A.G., Visser O., Posthuma E.F.M., Huijgens P.C., Sonneveld P., van de Loosdrecht A.A., Jongen-Lavrencic M. (2017). MDS classification is improving in an era of the WHO 2016 criteria of MDS: A population-based analysis among 9159 MDS patients diagnosed in the Netherlands. Cancer Epidemiol..

[18] Hellstrom-Lindberg E., Gulbrandsen N., Lindberg G., Ahlgren T., Dahl I.M., Dybedal I., Grimfors G., Hesse-Sundin E., Hjorth M., Kanter-Lewensohn L. (2003). A validated decision model for treating the anaemia of myelodysplastic syndromes with erythropoietin + granulocyte colony-stimulating factor: Significant effects on quality of life. Br. J. Haematol..

[19] Frosch Z.A., Abel G.A. (2016). Assessing Quality of Care for the Myelodysplastic Syndromes. Curr. Hematol. Malig. Rep..

[20] Lugtenberg M., Zegers-van Schaick J.M., Westert G.P., Burgers J.S. (2009). Why don’t physicians adhere to guideline recommendations in practice? An analysis of barriers among Dutch general practitioners. Implement. Sci..

[21] Kasprzak A., Nachtkamp K., Kondakci M., Schroeder T., Kobbe G., Kündgen A., Kaivers J., Rautenberg C., Haas R., Gattermann N. (2020). Analysis of the impact of adherence to guidelines and expert advice in patients with myelodysplastic syndromes. Ann Hematol..

[22] Harris P.A., Taylor R., Minor B.L., Elliott V., Fernandez M., O’Neal L., McLeod L., Delacqua G., Delacqua F., Kirby J. (2019). The REDCap consortium: Building an international community of software platform partners. J. Biomed. Inform..

[23] Arber D.A., Orazi A., Hasserjian R., Thiele J., Borowitz M.J., Le Beau M.M., Bloomfield C.D., Cazzola M., Vardiman J.W. (2016). The 2016 revision to the World Health Organization (WHO) classification of myeloid neoplasms and acute leukemia. Blood.

[24] Steensma D.P., Bejar R., Jaiswal S., Lindsley R.C., Sekeres M.A., Hasserjian R.P., Ebert B.L. (2015). Clonal hematopoiesis of indeterminate potential and its distinction from myelodysplastic syndromes. Blood.

[25] University of Chicago Hematopoietic Malignancies Cancer Risk Team (2016). How I diagnose and manage individuals at risk for inherited myeloid malignancies. Blood.

[26] Vardiman J.W., Thiele J., Arber D.A., Brunning R.D., Borowitz M.J., Porwit A., Harris N.L., Le Beau M.M., Hellstrom-Lindberg E., Tefferi A. (2009). The 2008 revision of the World Health Organization (WHO) classification of myeloid neoplasms and acute leukemia: Rationale and important changes. Blood.

[27] Bennett J.M., Catovsky D., Daniel M.T., Flandrin G., Galton D.A., Gralnick H.R., Sultan C. (1982). Proposals for the classification of the myelodysplastic syndromes. Br. J. Haematol..

[28] Ogata K., Della Porta M.G., Malcovati L., Picone C., Yokose N., Matsuda A., Yamashita T., Tamura H., Tsukada J., Dan K. (2009). Diagnostic utility of flow cytometry in low-grade myelodysplastic syndromes: A prospective validation study. Haematologica.

[29] Greenberg P.L., Tuechler H., Schanz J., Sanz G., Garcia-Manero G., Sole F., Bennett J.M., Bowen D., Fenaux P., Dreyfus F. (2012). Revised international prognostic scoring system for myelodysplastic syndromes. Blood.

[30] Greenberg P., Cox C., LeBeau M.M., Fenaux P., Morel P., Sanz G., Sanz M., Vallespi T., Hamblin T., Oscier D. (1997). International scoring system for evaluating prognosis in myelodysplastic syndromes. Blood.

[31] Malcovati L., Germing U., Kuendgen A., Della Porta M.G., Pascutto C., Invernizzi R., Giagounidis A., Hildebrandt B., Bernasconi P., Knipp S. (2007). Time-dependent prognostic scoring system for predicting survival and leukemic evolution in myelodysplastic syndromes. J. Clin. Oncol..

[32] Sorror M.L., Maris M.B., Storb R., Baron F., Sandmaier B.M., Maloney D.G., Storer B. (2005). Hematopoietic cell transplantation (HCT)-specific comorbidity index: A new tool for risk assessment before allogeneic HCT. Blood.

[33] Della Porta M.G., Malcovati L., Strupp C., Ambaglio I., Kuendgen A., Zipperer E., Travaglino E., Invernizzi R., Pascutto C., Lazzarino M. (2011). Risk stratification based on both disease status and extra-hematologic comorbidities in patients with myelodysplastic syndrome. Haematologica.

[34] Charlson M.E., Pompei P., Ales K.L., MacKenzie C.R. (1987). A new method of classifying prognostic comorbidity in longitudinal studies: Development and validation. J. Chronic Dis..

[35] Tong W.G., Quintas-Cardama A., Kadia T., Borthakur G., Jabbour E., Ravandi F., Faderl S., Wierda W., Pierce S., Shan J. (2012). Predicting survival of patients with hypocellular myelodysplastic syndrome: Development of a disease-specific prognostic score system. Cancer.

[36] Stojkov K., Silzle T., Stussi G., Schwappach D., Bernhard J., Bowen D., Cermak J., Dinmohamed A.G., Eeltink C., Eggmann S. (2020). Guideline-based indicators for adult patients with myelodysplastic syndromes. Blood Adv..

[37] Platzbecker U., Symeonidis A., Oliva E.N., Goede J.S., Delforge M., Mayer J., Slama B., Badre S., Gasal E., Mehta B. (2017). A phase 3 randomized placebo-controlled trial of darbepoetin alfa in patients with anemia and lower-risk myelodysplastic syndromes. Leukemia.

[38] Platzbecker U., Wong R.S., Verma A., Abboud C., Araujo S., Chiou T.J., Feigert J., Yeh S.P., Gotze K., Gorin N.C. (2015). Safety and tolerability of eltrombopag versus placebo for treatment of thrombocytopenia in patients with advanced myelodysplastic syndromes or acute myeloid leukaemia: A multicentre, randomised, placebo-controlled, double-blind, phase 1/2 trial. Lancet Haematol..

[39] Giagounidis A., Mufti G.J., Fenaux P., Sekeres M.A., Szer J., Platzbecker U., Kuendgen A., Gaidano G., Wiktor-Jedrzejczak W., Hu K. (2014). Results of a randomized, double-blind study of romiplostim versus placebo in patients with low/intermediate-1-risk myelodysplastic syndrome and thrombocytopenia. Cancer.

[40] Kantarjian H.M., Fenaux P., Sekeres M.A., Szer J., Platzbecker U., Kuendgen A., Gaidano G., Wiktor-Jedrzejczak W., Carpenter N., Mehta B. (2018). Long-term follow-up for up to 5 years on the risk of leukaemic progression in thrombocytopenic patients with lower-risk myelodysplastic syndromes treated with romiplostim or placebo in a randomised double-blind trial. Lancet Haematol..

[41] Blumenberg C., Barros A.J.D. (2018). Response rate differences between web and alternative data collection methods for public health research: A systematic review of the literature. Int. J. Public Health.

[42] Pease D.F., Ross J.A., Poynter J.N., Nguyen P.L., Hirsch B., Cioc A., Roesler M.A., Warlick E.D. (2015). Differences in community and academic practice patterns for newly diagnosed myelodysplastic syndromes (MDS) patients. Cancer Epidemiol..

[43] Boulkedid R., Abdoul H., Loustau M., Sibony O., Alberti C. (2011). Using and reporting the Delphi method for selecting healthcare quality indicators: A systematic review. PLoS ONE.

[44] Barth J.H., Misra S., Aakre K.M., Langlois M.R., Watine J., Twomey P.J., Oosterhuis W.P. (2016). Why are clinical practice guidelines not followed?. Clin. Chem. Lab. Med..

[45] Lawrence M., Olesen F. (1997). Indicators of Quality in Health Care. Eur. J. Gen. Pract..

[46] Porter M.E. (2010). What is value in health care?. N. Engl. J. Med..

[47] Campbell S.M., Braspenning J., Hutchinson A., Marshall M. (2002). Research methods used in developing and applying quality indicators in primary care. Qual. Saf. Health Care.

[48] Kotter T., Blozik E., Scherer M. (2012). Methods for the guideline-based development of quality indicators—A systematic review. Implement. Sci..

