# Supplementary material for: Survey on Recommended Health Care for Adult Patients with Myelodysplastic Syndromes Identifies Areas for Improvement
Source: Int J Environ Res Public Health. 2020 Dec 21;17(24):9562. doi: 10.3390/ijerph17249562 (PMC7766925; doi:10.3390/ijerph17249562)
Supplement: Supplementary file 1 [file ijerph-17-09562-s001.pdf]

Supplemental table 1: Questions with insufficient feedbacks.

Questions	Feedback
**1. Diagnostic evaluation**	
**Molecular diagnostics**	
Please specify the single genes you screen	3 (7.0%)
**2. Risk-stratification**	
**Other**	
Which geriatric assessment tools do you use?	12 (27.9%)
Which quality of life (QoL) assessment tools do you use?	5 (11.6%)
**3. Supportive care**	
**Infectious prophylaxis**	
Do you provide primary anti-bacterial prophylaxis?	11 (25.6%)
What is your preferred compound for primary anti-bacterial prophylaxis?	3 (7.0%)
Which other compound(s) do you use for anti-bacterial prophylaxis?	0
Do you provide primary anti-mycotic prophylaxis?	11 (25.6%)
What is your preferred compound for primary anti-mycotic prophylaxis?	8 (18.6%)
Which other compound(s) do you use for primary anti-mycotic prophylaxis?	0
Do you provide primary anti-viral prophylaxis?	11 (25.6%)
What is your preferred compound for primary anti-viral prophylaxis?	6 (14%)
Which other compound(s) do you use for primary anti-viral prophylaxis?	0
Do you provide primary anti-Pneumocystis prophylaxis?	11 (25.6%)
What is your preferred compound for anti-PCP prophylaxis?	6 (14%)
Do you use a CD4 T-cell lymphocyte trigger to provide anti-viral and anti-PCP prophylaxis?	6 (14%)
What is the CD4 threshold value (cells/µl) you use?	1 (2.3%)
**4. Growth factors**	
**Erythropoietin Stimulating Agents (ESA)**	
What is your initial starting dose for epoetin beta (e.g. Recormon®, total dose/Units)/epoetin alfa (e.g. Eprex®, total dose/Units) or darbepoetin alfa (Aranesp®, total dose/mcg) in patients without renal failure?	0
What is your initial dosing interval?	0
What is the dosing schedule you use for G-CSF treatment in ESA-refractory patients?	10 (23.3%)
**Granulocyte-Colony-Stimulating Factor (G-CSF)**	
What is your neutrophil trigger for primary infection prophylaxis?	4 (9.3%)
**Thrombopoietin-Receptor Agonist (TPO-RA)**	
Which other dose do you use?	2 (4.7%)
**5. Disease modifying treatment**	
**Lenalidomid (LEN)**	
Which thromboembolic prophylaxis do you use in patients with sufficient platelet count?	11 (25.6%)
Specify the drug you use for thromboembolic prophylaxis	1 (2.3%)
**Immunosuppressive treatment (IST)**	
What type of ATG do you use?	2 (4.7%)
What CsA serum level do you aim for, if tolerated?	2 (4.7%)
How long do you wait for response to ATG/CsA (months)?	2 (4.7%)
How many ATG cycles do you try?	2 (4.7%)
What is your indication for steroids?	14 (22.6%)
**Hypomethylating agents (HMA)**	
Please specify the standard dose you use for 5-azacytidine (AZA)	0
What is the standard dose you use for decitabine (DEC)?	1 (2.3%)
Please specify the standard dose you use for decitabine (DEC).	0
To which extent do you reduce the dose of HMA in responding patients after 6 cycles?	0
How do you extend the interval of HMA application in responding patients after 6 cycles?	9 (20.9%)
**6. Allogeneic hematopoietic stem cell transplantation (n=6)**	
**Induction therapy**	

Please specify your preferential induction chemotherapy regimen for fit MDS patients with <5% blasts eligible for allo HSCT	0
Please specify your preferential induction chemotherapy regimen for fit MDS patients with >5% blasts eligible for allo HSCT	0
Please specify your preferential induction chemotherapy regimen for unfit MDS patients with <5% blasts eligible for allo HSCT	0
Please specify your preferential induction chemotherapy regimen for less fit MDS patients with >5% blasts eligible for allo HSCT	0
**Conditioning therapy**	
Please specify your preferred conditioning regimen for fit MDS patients with <5% BM blasts	0
Please specify your preferred conditioning regimen for fit MDS patients with >5% BM blasts	0
Please specify your preferred conditioning regimen for less fit MDS patients with <5% BM blasts	0
Please specify your preferred conditioning regimen for less fit MDS patients with >5% BM blasts	0
